# Bioactive Potential of “Jaspeado Garlic” (*Allium sativum*) from Northwestern Mexico: In Vitro Antioxidant and Bacteriostatic Effects

**DOI:** 10.3390/metabo16080572

**Published:** 2026-08-12

**Authors:** Elizabeth Varela-Navarro, Luis Alberto Anguiano-Sevilla, Gilberto Velázquez-Juárez, Ivan David Meza-Canales, Rocío Ivette López-Roa, María Eugenia Jaramillo-Flores, Fabián Rho-Mas, Julio César Muro-Valdez, Abril Melchor-González, Adelaida Sara M. Zepeda-Morales

**Affiliations:** 1 Laboratorio de Análisis Clínicos e Investigación Traslacional, Departamento de Farmacobiología, Centro Universitario de Ciencias Exactas e Ingenierías, Universidad de Guadalajara, Guadalajara 44430, Mexico; elizabeth.varela@academicos.udg.mx (E.V.-N.); alberto.anguiano@academicos.udg.mx (L.A.A.-S.); julio.muro@academicos.udg.mx (J.C.M.-V.); abril.melchor4890@alumnos.udg.mx (A.M.-G.); 2 Laboratorio de Bioquímica Estructural, Departamento de Química, Centro Universitario de Ciencias Exactas e Ingenierías, Universidad de Guadalajara, Guadalajara 44430, Mexico; gilberto.velazquez@academicos.udg.mx; 3 Laboratorio de Investigación y Desarrollo Farmacéutico, Departamento de Farmacobiología, Centro Universitario de Ciencias Exactas e Ingenierías, Universidad de Guadalajara, Guadalajara 44430, Mexico; ivan.meza5024@academicos.udg.mx (I.D.M.-C.); rocio.lopez@academicos.udg.mx (R.I.L.-R.); 4 Laboratorio de Biopolímeros, Departamento de Ingeniería Bioquímica, Escuela Nacional de Ciencias Biológicas, Instituto Politécnico Nacional IPN, Alcaldía Gustavo A. Madero, Ciudad de Mexico 07738, Mexico; jaramillo_flores@hotmail.com; 5 School of Medicine and Health Sciences, Tecnologico de Monterrey, Guadalajara Campus, Zapopan 45138, Mexico; fabianrhomas@tec.mx

**Keywords:** garlic, alliin, antioxidant activity, antimicrobial activity, minimum inhibitory concentration, 3T3-L1 adipocytes

## Abstract

**Background/Objectives:** Garlic (*Allium sativum*) has long been valued for its rich constellation of organosulfur and phenolic compounds, molecules that underpin its many recognized biological activities. Despite its deep roots in traditional medicine, the functional profile of garlic is far from uniform; it shifts notably depending on how its bioactive components are extracted. Most scientific attention has gravitated toward ethanolic extracts, leaving the behavior of aqueous-buffer preparations relatively unexplored. In this study, we evaluated the antioxidant, antimicrobial, and exploratory immunometabolic effects of extracts obtained with phosphate-buffered saline (PBS) and 50% ethanol in PBS (EtOH:PBS; 50:50, *v*/*v*), using a regional garlic variety (“Jaspeado”) cultivated in northwestern Mexico. **Methods:** Their antioxidant potential was profiled through DPPH, ABTS, and ORAC assays; phenolic content was quantified using the Folin–Ciocalteu method, and a broader chemical fingerprint was obtained through UPLC-ESI-TQ-MS/MS. Furthermore, we analyzed their antimicrobial action against *Escherichia coli*, *Salmonella* spp., and *Staphylococcus aureus*, and examined their immunometabolic influence in LPS-stimulated 3T3-L1 adipocytes. **Results:** The extracts showed distinct, solvent-dependent patterns of antioxidant activity. The PBS extract displayed the highest ORAC values, whereas the EtOH:PBS extract showed a stronger DPPH and ABTS activity and a higher phenolic recovery. The PBS extract exhibited a broader bacteriostatic effect, inhibiting the growth of all strains, and selectively modulated antioxidant gene expression and influenced cytokine changes in adipocytes. **Conclusions:** Overall, this regional garlic variety exhibits solvent-dependent antioxidant and antimicrobial activities, while the PBS extract provided a biocompatible preparation for exploring relationships between chemical composition and biological activities.

## 1. Introduction

Natural bioactive compounds, such as plant-derived extracts, have shown promise in diverse health conditions, including those associated with oxidative stress, chronic inflammation, and microbial imbalance [1,2]. These conditions are characterized by cytokine imbalance, and loss of microbial homeostasis, which together favor metabolic dysfunction [2,3]. This highlights the need for bioactive compounds capable of restoring redox equilibrium and controlling microbial overgrowth without promoting antibiotic resistance.

The genus *Allium* has garnered increasing attention because of its broad biological and therapeutic potential [1]. Comprising more than 700 species distributed worldwide, plants of the genus *Allium* have been traditionally used both as food and in medicine [4]. Among them, garlic (*Allium sativum*) stands out for its rich constellation of active secondary metabolites, such as organosulfur compounds (OSCs) and phenols, which are associated with antioxidant, antimicrobial, and immunomodulatory effects [5,6]. These bioactive molecules, mainly organosulfur compounds derived from cysteine metabolism, contribute to the biological activities of garlic by modulating cellular redox homeostasis and inhibiting microbial growth [1,4,7]. Alliin (S-allyl-L-cysteine sulfoxide) requires mechanical disruption of garlic tissue for its enzymatic conversion, because alliinase becomes active only when the tissue is crushed or chopped and both components mix. This reaction rapidly transforms alliin into allicin, thiosulfinates and other sulfur compounds responsible for many of garlic’s biological effects [8,9]. However, alliin content does not directly reflect allicin formation, because allicin generation depends on alliinase activity, tissue disruption, reaction conditions, while allicin is also subject to rapid chemical degradation. Allicin was not directly quantified in the present study. The antimicrobial effects of garlic extend to Gram-positive and Gram-negative bacteria, including *Staphylococcus aureus* and *Escherichia coli* [10,11,12]. At a cellular level, allicin and related OSCs modify protein thiols, disrupt bacterial membranes, and impair metabolic enzymes [11]. Synergistic interactions between allicin and antibiotics have also been reported [10]. Beyond their antimicrobial effects, garlic and its derivatives exhibit notable antioxidant properties, commonly evaluated using the 2,2-diphenyl-1-picrylhydrazyl (DPPH) radical scavenging assay, the 2,2′-azino-bis (3-ethylbenzothiazoline-6-sulfonic acid) (ABTS) radical cation scavenging assay, and the oxygen radical absorbance capacity (ORAC) assay [13,14]. Whereas DPPH and ABTS primarily assess electron-transfer and general radical-scavenging activity, ORAC specifically quantifies peroxyl radical quenching, a process highly relevant to biological systems [13,15].

Extraction solvent composition strongly determines the metabolite profile recovered. Hydroalcoholic systems enhance phenolic extraction [16], whereas PBS-based extraction may favor the stability of water-soluble organosulfur compounds and provides a biocompatible, pH-stabilizing and biologically inert medium suitable for cellular applications [9,17]. Importantly, organic solvents often require additional removal steps that may result in loss or degradation of volatile or labile OSCs, whereas PBS enables direct downstream biological use without preprocessing, an advantage for studies focusing on immunometabolic responses [17,18]. High-performance liquid chromatography (HPLC) is commonly used to quantify alliin as a marker of garlic quality [19], while UPLC-MS/MS allows the elucidation of the bioactive compounds profiles related to the solvent used for extraction [20,21,22].

In addition to antimicrobial and antioxidant properties, recent studies emphasize the influence of garlic and its derivatives on cellular signaling related to oxidative stress and inflammation. In 3T3-L1 adipocytes, alliin and garlic extracts modulate adipogenic and inflammatory pathways by regulating genes such as PPAR-γ, C/EBPα, and antioxidant enzymes [3,7,23]. Garlic-derived compounds have been shown to reduce pro-inflammatory cytokines (IL-6, TNF-α) and promote anti-inflammatory mediators (IL-10), linking their antioxidant actions to immunometabolic regulation [3].

Despite extensive studies on garlic, most use ethanolic or methanolic extracts, and very few have explored alternative solvents such as phosphate-buffered saline (PBS). PBS allows its direct use in in vitro systems without compromising cell viability, as well as potential applicability in vivo. Importantly, solvent composition favors different metabolite classes and alters biological properties [24].

In addition, regional garlic cultivars remain insufficiently characterized despite their agricultural importance and potentially distinctive chemical profiles. The “Jaspeado” garlic grown in northwestern Mexico offers a model to study how solvent-dependent extraction influences the recovery of bioactive compounds and their biological activities.

In this study, we aimed to evaluate the biological potential of the PBS extract by comparing its antioxidant, antimicrobial, and cytokine-modulating properties. We characterized its antioxidant and antimicrobial capacities and investigated its effects on LPS-stimulated 3T3-L1 adipocytes, focusing on antioxidant gene expression and exploratory cytokine modulation. This approach provides an integrative framework linking chemical composition to biological response, bridging antioxidant and antimicrobial actions with immunometabolic regulation. By combining a regional garlic variety with a biocompatible PBS extraction system, this work brings attention to aspects relevant for understanding garlic bioactivity.

## 2. Materials and Methods

### 2.1. Plant Material

The plant material consisted of bulbs of the garlic (*A. sativum*) cultivar “Jaspeado” (a.k.a. purple garlic), a Korean-origin variety adapted for cultivation in Mexico and traditionally grown in the central–northwestern region of the country [25]. Bulbs were kindly provided by Agrícola Los Rancheros S.A. de C.V. (San Marcos, Aguascalientes, Mexico). Taxonomic identification was conducted using morphological characteristics, dichotomous keys, and comparison with specimens from the Luz María Villarreal de Puga Herbarium (University of Guadalajara), as well as with the databases International Plant Names Index, World Checklist of Selected Plant Families, and The Plant List, following the APG IV classification system. The bulbs were manually harvested in May 2021 at commercial maturity (0.2–0.3% bulb ratio and 38–50% foliar senescence) in El Llano locality in Aguascalientes, México. After harvest, they were cured under ambient conditions (30–35 °C, 65–75% relative humidity), and only undamaged bulbs with uniform size and weight were selected [26]. Bulbs were then cryo-ground using dry ice and stored at −80 °C until use.

### 2.2. Extraction and Yield Estimation

Extraction was performed following the method of Nagella et al. [5] with some modifications. Fresh garlic bulbs were cryo-ground and extracted by maceration at room temperature in the dark for 24 h at 1:10 (*w*/*v*) ratio using one of two extraction systems: phosphate-buffered saline (PBS) or 50% ethanol in PBS (EtOH:PBS; 50:50, *v*/*v*). PBS was prepared at 0.1 M and used either as the sole extraction solvent or as the aqueous phase of the EtOH:PBS system.

After extraction, the PBS and EtOH:PBS extracts were filtered using filter paper and ethanol was removed from the EtOH:PBS extracts by rotary evaporation (Büchi I100). Both extracts were then freeze-dried under standard lyophilization conditions (−50 °C, <0.1 mbar, 20–30 h; Labconco Freeze Dryer 4.5) and stored at −80 °C until analysis. Extraction yield was calculated as the ratio of the garlic lyophilized weight (LW) to the initial fresh mass of the garlic, using the following formula.

$$Yield\;(\%)=\left(\frac{Extract\;lyophilized\;weight\;(LW)}{\;Initial\;fresh\;weight}\right)\times 100\;$$

### 2.3. Antioxidant Capacity and Total Polyphenol Determination

Antioxidant capacity was evaluated using three complementary assays: the DPPH (2,2-diphenyl-1-picrylhydrazyl) radical scavenging assay, the ABTS (2,2′-azino-bis (3-ethylbenzothiazoline-6-sulfonic acid)) radical cation scavenging assay, and the ORAC (oxygen radical absorbance capacity) assay, as described below.

#### 2.3.1. DPPH Radical Scavenging Assay

The antioxidant activity was determined by the ability of the samples to scavenge the stable radical DPPH. In a 96-well microplate, 20 µL of sample, blank, or Trolox standard (0–500 µM) was mixed with 180 µL of DPPH solution (150 µM in methanol:water, 80:20 *v*/*v*). The plate was incubated in the dark at room temperature for 30 minmin, and absorbance was measured at 515 nm. Results were expressed as micromoles of Trolox equivalents (µmol TE) per gram of garlic LW [27].

#### 2.3.2. ABTS Radical Cation Scavenging Assay

The ABTS radical cation (ABTS^•+^) was generated by mixing a 7 mM ABTS stock solution with 2.4 mM potassium persulfate (K_2_S_2_O_8_) and allowing the mixture to react for 16 h in the dark at room temperature. The working solution was prepared by diluting 1 mL of the stock with 45 mL of distilled water to achieve an absorbance of 0.70 ± 0.02 at 734 nm. For the assay, 20 µL of each sample, blank, or Trolox standard (0–500 µM) was added to a 96-well plate, followed by 280 µL of the ABTS working solution. After incubation in the dark for 30 min at room temperature, absorbance was recorded at 754 nm. Results were reported as µmol TE per gram of garlic LW [28].

#### 2.3.3. Oxygen Radical Absorbance Capacity (ORAC) Assay

The ORAC assay was performed to evaluate antioxidant capacity based on the inhibition of peroxyl-radical-induced oxidation. In a black 96-well microplate, 20 µL of diluted sample or Trolox standard (0–50 µM) was combined with 120 µL of fluorescein solution (96 nM in 75 mM phosphate buffer, pH 7.4). After a 20 min incubation at 37 °C, the reaction was initiated by adding 60 µL of AAPH (12 mM). Fluorescence was measured every 2 min for 120 min using a Synergy H1 microplate reader (Biotek Instruments, Inc., Winooski, VT, USA), with excitation and emission wavelengths set at 485/20 nm and 528/25 nm, respectively. Antioxidant activity was quantified as the area under the fluorescence decay curve (AUC), and results were expressed as µmol TE per milligram of garlic LW [15].

### 2.4. Determination of Total Polyphenol Content Using the Folin–Ciocalteu Method

Total polyphenol content was determined using the Folin–Ciocalteu colorimetric assay. A standard curve of gallic acid (0–0.1 mg GAE/mL) was prepared in a 96-well microplate. For each reaction, 20 µL of sample, blank, or gallic acid standard was added to a well, followed by 100 µL of diluted Folin–Ciocalteu reagent (1:4 dilution in distilled water) and 75 µL of 10% (*w*/*v*) sodium carbonate (Na_2_CO_3_). Plates were incubated in the dark at room temperature for 2 h. Absorbance was measured at 750 nm. Results were expressed as milligrams of gallic acid equivalents (mg GAE) per gram of garlic LW [27].

### 2.5. Chromatographic Conditions for Alliin Detection and Quantification

Alliin was quantified using an Agilent 1120 HPLC system equipped with a UV-VIS detector and a Zorbax SB-C18 column (5 µm, 150 × 4.6 mm). The mobile phase consisted of ultrapure water acidified with formic acid (pH 5.2) as solvent A and methanol as solvent B, in an 80:20 (*v*/*v*) ratio. The isocratic elution was maintained for 9 min at a flow rate of 0.3 mL/min. The injection volume was 10 µL, and detection was performed at 214 nm. Standard solutions of alliin were prepared in HPLC-grade water, and a calibration curve was generated in the range of 25–150 ppm [19].

### 2.6. UPLC-ESI-TQ-MS/MS Analysis

Ultra-performance liquid chromatography coupled to electrospray ionization triple-quadrupole tandem mass spectrometry (*UPLC-ESI-TQ-MS/MS*) was performed with modifications based on Hsieh-Lo et al. [29], using a Waters Acquity UPLC system (Waters Corporation, Milford, MA, USA) equipped with a flow-through needle autosampler and coupled to a Xevo TQ triple quadrupole mass spectrometer. Chromatographic separation was achieved on a BEH C18 column (1.7 µm, 2.1 × 50 mm) maintained at 40 °C, with a flow rate of 0.3 mL/min. The mobile phases consisted of acetonitrile with 0.1% formic acid (solvent A) and water with 0.1% formic acid (solvent B), using the following gradient: 5% A, 0–1 min; 10% A, 2 min; 50% A, 8 min; 70% A, 12 min; 95% A, 14 min; and 5% A at 16 min for re-equilibration.

Mass spectrometric detection was performed using electrospray ionization in positive ion mode (ESI+), with a capillary voltage of 3.0 kV, cone voltage of 30 V, source temperature of 150 °C, and desolvation temperature of 200 °C. When applied, the collision energy was set to 40 eV. Data were acquired in full-scan MS mode over an *m*/*z* range of 50–1200, or using MRM and daughter ion scan modes for specific precursor ions. This acquisition strategy was selected to obtain an overall comparative chemical profile of the PBS and EtOH:PBS extracts. Putative metabolite annotations were based on precursor *m*/*z*, retention time (RT), comparisons with database and published literature and, for selected ions, MS/MS fragmentation. Because these UPLC-MS/MS-based metabolites annotations were not confirmed with authentic standards, they should be considered tentative.

Data acquisition and processing were performed using MassLynx software v4.1 SCN 945 with SCN 960 (Waters Corporation, Milford, MA, USA), spectral visualization and inspection were carried out using MestReNova (v. 12.0.0-20080; Mestrelab Research S.L., A Coruña, Spain), and further data processing, statistical analyses, and visualization were performed in R (v. 4.5.3; R Core Team, Vienna, Austria).

### 2.7. Antimicrobial Activity and Minimum Inhibitory Concentration

Antimicrobial activity was assessed following the method described by Dueñas-Bolaños et al. [30], using reference bacterial strains: *E. coli* ATCC 25922, *Salmonella* spp., and *S. aureus*. Bacteria were cultured on tryptic soy agar and incubated at 37 °C for 24 h prior to testing.

The minimum inhibitory concentration (MIC) was determined using standard clinical laboratory protocols. Bacterial suspensions were adjusted to a turbidity equivalent to a 0.5 McFarland standard (~10^8^ CFU/mL), then diluted to achieve a final concentration of 10^5^ CFU/mL. Ampicillin (1280 µg/mL) was used as growth-inhibition control, while untreated bacterial cultures served as growth controls.

The MIC assay was conducted in a 96-well microdilution format. Each well was filled with 100 µL of Mueller-Hinton broth (MH). In the first well of each dilution series, 100 µL of either the PBS or EtOH:PBS extract (60 mg/mL) was added, followed by serial twofold dilutions across the remaining wells. Subsequently, 10 µL of the bacterial inoculum was added to each well. Plates were incubated at 37 °C for 24 h, and bacterial growth was monitored by measuring optical density at 620 nm (OD_620_) using a UV-Vis spectrophotometer. The MIC was defined as the lowest concentration of the tested extract that resulted in a significant reduction in OD_620_, indicating inhibition of bacterial growth [30].

Bactericidal activity was defined according to standard criteria as a ≥99.9% reduction in viable bacterial counts, equivalent to a ≥3 log_10_ decrease in CFU, which is considered the minimum threshold to classify an agent as bactericidal [31].

### 2.8. 3T3-L1 Cell Culture and Adipogenic Differentiation

Adipogenic differentiation of 3T3-L1 preadipocytes was carried out following the protocol by Quintero-Fabián et al. [3], with minor modifications. 3T3-L1 cells (ATCC, Manassas, VA, USA) were cultured in high-glucose Dulbecco’s Modified Eagle Medium (DMEM, Merck, Darmstadt, Germany) supplemented with 10% fetal bovine serum (FBS) and 1% penicillin–streptomycin until reaching full confluence. Two days after confluence, differentiation was initiated using the 3T3-L1 Differentiation Kit (Cat. No. DIF001, Merck, Germany), in accordance with the manufacturer’s instructions. The differentiation kit cocktail contained 1.5 µg/mL insulin, 1 µM dexamethasone, 500 µM 3-isobutyl-1-methylxanthine, and 1 µM rosiglitazone, and was applied for 72 h. Following induction, cells were maintained in DMEM supplemented with 10% FBS and 1.5 µg/mL insulin, with medium renewal every 2–3 days for up to 8 days. After the differentiation, and prior to the gene expression and cytokine analyses, cells were serum-deprived (0% FBS) for 24 h to avoid further growth. For both, expression and cytokine analyses, cells were treated with LPS (100 ng/mL). Experimental groups included untreated control cells, LPS-treated cells (+LPS), and LPS-treated cells exposed to either PBS extract at alliin-equivalent concentrations (0.1 or 0.5 mM) or pure alliin (0.1 or 0.5 mM). The dose calculation is presented in Supplementary S2 on the Supplementary Materials according to Quintero-Fabián [3,32].

### 2.9. Gene Expression of Antioxidant Biomarkers

The expression of genes encoding antioxidant enzymes, Catalase (*CAT*), superoxide dismutase (*SOD*), and glutathione peroxidase (*GPx*), was analyzed by RT-PCR [3]. Total RNA was isolated using TRIzol™ Reagent (Invitrogen, Thermo Fisher Scientific, Waltham, MA, USA; Cat. 15596026) following the manufacturer’s protocol. Briefly, cells were homogenized in TRIzol, phase separation was performed with chloroform, the aqueous phase was precipitated with isopropanol, the RNA pellet was washed with 75% ethanol, and resuspended in RNase-free water. RNA concentration and purity were assessed by A260/A280.

Complementary DNA (cDNA) was synthesized from 3 µg of total RNA using the iScript™ cDNA Synthesis Kit for RT-qPCR (Bio-Rad Laboratories, Inc., Hercules, CA, USA; Cat. 1708891), according to the manufacturer’s instructions.

Gene expression was quantified by RT-qPCR with SYBR Green chemistry, SsoAdvanced Universal SYBR^®^ Green Supermix (Bio-Rad Laboratories, Inc., Hercules, CA, USA; Cat. 1725270), on a Rotor-Gene Q cycler (Qiagen, Hilden, Germany). Primers were designed with Primer-BLAST and are listed in Table 1. *Rps18s* served as the reference gene.

Each 10 µL reaction contained 5 µL SYBR Green mix, 0.4 µL of each primer (forward and reverse), 2.2 µL nuclease-free water, and 2 µL cDNA. Cycling conditions were: 95 °C for 30 s; 45 cycles of 95 °C for 15 s, gene-specific annealing for 15 s (temperatures in Table 1), and—only for amplicons > 150 bp—72 °C for 30 s; followed by a melt-curve ramp from 45 °C to 95 °C. Relative mRNA levels were calculated by the 2^−ΔΔCt^ method using Rps18s for normalization. Results are reported as mean ± SD [7].

### 2.10. Cytokine Analysis

Cytokine levels were detected by using the MULTI-PLEX technique with the Bio-Plex Pro^TM^ Mouse Cytokine Th17 Panel A 6-Plex Kit^®^ (Bio-Rad Laboratories, Inc., Hercules, CA, USA; Cat. M6000007NY), following the manufacturer’s instructions. Before analysis, samples were diluted 1:4 by mixing 40 μL of cell supernatant with 120 μL cell culture media.

### 2.11. Statistical Analysis

For all the data normality was assessed using the Shapiro–Wilk normality test, and homogeneity of variance was verified with Bartlett’s test. If assumptions were met, group differences were analyzed by one-way ANOVA followed by Tukey’s post hoc test and Welch’s *t*-test. In cases where assumptions were not satisfied, a generalized linear model (GLM) with an appropriate distribution was applied, and model adequacy was confirmed by inspection of deviance residuals and a Shapiro–Wilk test of residuals.

Chemical and microbiological assays were performed using a single extract preparation for each solvent condition. The same extract preparation was evaluated in three separate analytical runs, with technical triplicates performed within each run. Technical replicates were averaged for each analytical run. Thus, *n* = 3 represents three analytical runs of the same extract preparation and not three independently prepared extracts.

For gene expression analysis, nine wells per condition were used within a single cell-culture experiment, and each well received the assigned treatment separately. After treatment, cells from three wells were pooled to generate one experimental sample, resulting in three pooled samples per condition (*n* = 3 pooled samples). RNA was extracted separately from each pooled sample, and the corresponding cDNA was analyzed in technical duplicate by RT-qPCR. Technical duplicate Ct values were averaged before relative gene expression analysis. Thus, *n* = 3 represents three pooled within-experiment samples and does not represent three independent cell-culture experiments or biological replicates performed on different days. Gene expression levels were normalized to *Rps18s* and calculated relative to the untreated control using the 2^−ΔΔCt^ method.

Cytokine measurements were obtained from three pooled cell-culture supernatants per condition within a single cell culture experiment. Each pooled sample was generated by combining the supernatants from three separately treated wells, corresponding to nine wells per condition. Each pooled sample was analyzed once with no technical replicates, and the fluorescence values obtained were averaged. Thus, the cytokine measurements were analyzed descriptively as an exploratory dataset. No inferential statistical testing or correction for multiple comparisons was applied across cytokines or treatment groups; therefore, the observed cytokine patterns were interpreted as hypothesis-generating rather than as confirmatory evidence of treatment effects.

Statistical analyses were performed using GraphPad Prism version 6.0 (GraphPad Software, San Diego, CA, USA) and R version 4.5.3. Unless otherwise specified, nominal statistical significance was defined as *p* < 0.05.

## 3. Results

### 3.1. Extraction Yield of PBS and EtOH:PBS Extracts

To compare the extraction yields obtained with PBS and 50% ethanol in PBS (EtOH:PBS; 50:50, *v*/*v*), the recovery of freeze-dried material was quantified for each extraction system. Extraction yield was higher for PBS (24.7 ± 4.1%) than for EtOH:PBS (17.9 ± 2.6%), corresponding to a difference of 6.8 percentage points (Table 2). The lower yield obtained with EtOH:PBS may be partly related to losses during ethanol removal. Solvent composition may also influence the chemical composition and biological properties of the extracts.

### 3.2. Comparative Antioxidant Capacity of Garlic Extracts Obtained with PBS and EtOH:PBS

**Figure 1:**
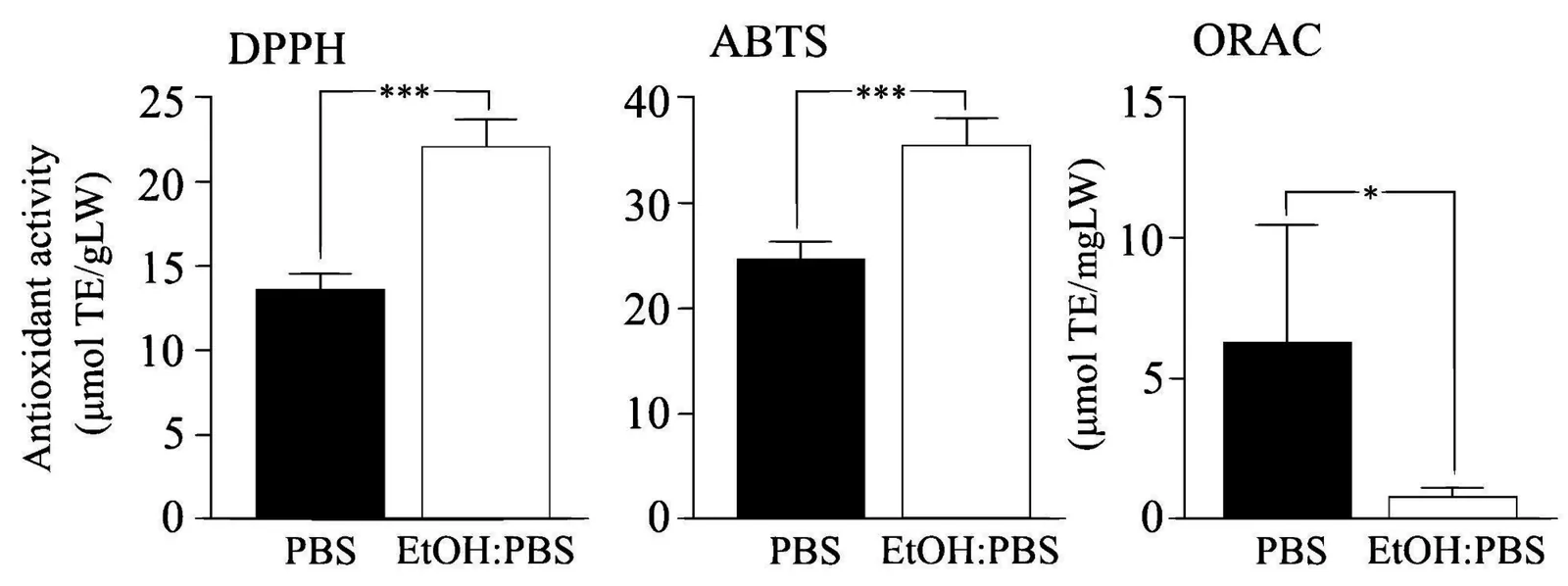
Antioxidant capacity of the PBS and EtOH:PBS extracts. Radical scavenging activity was assessed using DPPH and ABTS assays, while oxygen radical absorbance capacity was assessed by ORAC. Data are presented as mean ± SD from three analytical runs performed using the same extract preparation (*n* = 3 analytical runs). Each analytical run included technical triplicates. DPPH and ABTS results are expressed as µmol TE/g LW, whereas ORAC results are expressed as µmol TE/mg LW. TE, Trolox equivalents; LW, lyophilized garlic weight. Statistical differences were analyzed by ANOVA (DPPH and ABTS) or GLM (ORAC). Asterisks indicate statistically significant differences between extracts: * *p* < 0.05; *** *p* < 0.001. Calibration curves showed good linearity, with R^2^ values of 0.997 for DPPH, 0.998 for ABTS, and 0.993 for ORAC (Figure S1).

To further evaluate the properties of the extracts, we analyzed the antioxidant activity of the PBS and EtOH:PBS extracts using DPPH, ABTS, and ORAC assays. Both extracts exhibited radical scavenging capacity (Figure 1), with EtOH:PBS extract consistently showing higher activity in DPPH and ABTS assays, but not in ORAC. In the DPPH assay, the EtOH:PBS extract displayed a scavenging activity of 22.05 (±1.62) µmol TE/g lyophilized garlic Weight (LW), compared to 13.66 (±0.893) µmol TE/g LW for the PBS extract, approximately 1.6-fold higher. Similarly, in the ABTS assay, the EtOH:PBS extract reached 33.04 (±1.638) µmol TE/g LW, versus 24.84 (±1.436) µmol TE/g LW for PBS, about 1.3-fold higher.

Interestingly, the ORAC assay revealed a markedly higher antioxidant capacity in PBS extract (6.35 µmol TE/mg LW), approximately 7.7-fold higher than that of the EtOH:PBS extract (0.82 µmol TE/mg LW). The ORAC test monitors the inhibition of physiologically relevant peroxyl radicals over time, providing insight into the potential antioxidant activity under conditions that mimic oxidative stress in vivo. These results suggest that PBS extraction favors recovery of compounds with radical scavenging capacity under physiologically relevant conditions.

### 3.3. Total Polyphenol Content Determination

**Figure 2:**
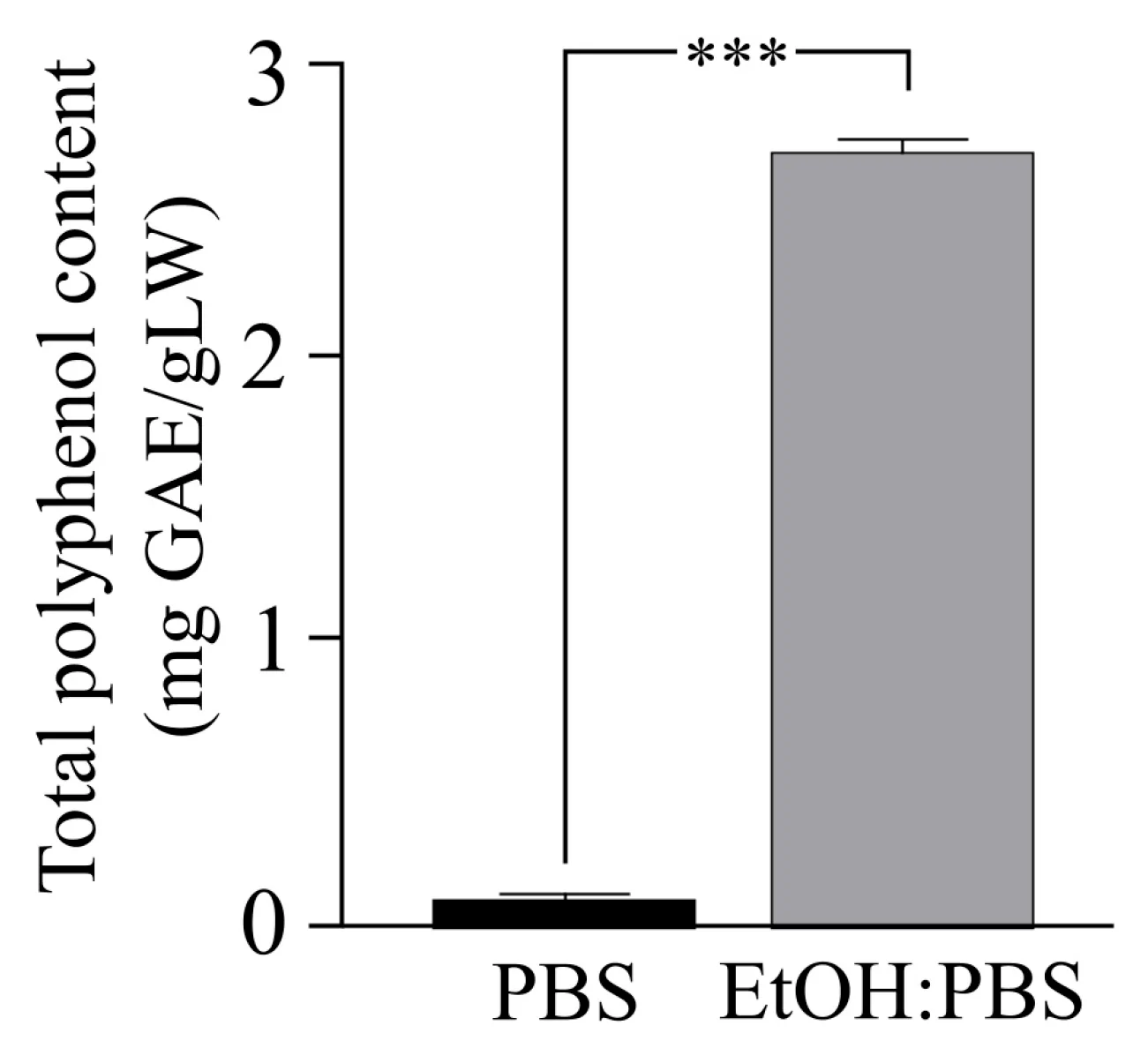
Total polyphenol content of the PBS and EtOH:PBS extracts. Polyphenol content was determined using the Folin–Ciocalteu method. Results are expressed as mean ± standard deviation in mg gallic acid equivalents (GAE) per gram of lyophilized garlic weight (g LW). Data are presented as mean ± SD from three analytical runs performed using the same extract preparation (*n* = 3 analytical runs). Each analytical run included technical triplicates. Statistical differences were assessed by Welch’s *t*-tests. Asterisks indicate statistically significant differences between extracts: *** *p* < 0.001. Calibration curve R^2^ = 0.998 (Figure S1).

Many metabolites contribute to antioxidant activity, with polyphenols being among the most important groups. To explore the relationship between total polyphenol content (TPC) and the previously observed antioxidant activity, TPC was quantified as mg of gallic acid equivalents (GAE) per g of lyophilized garlic weight (LW) (Figure 2). TPC ranged from 0.03 to 2.69 mg GAE/g LW, with the EtOH:PBS extract showing the highest concentration (2.699 ± 0.041 mg GAE/g LW), over 25-fold higher than the PBS extract (0.104 ± 0.018 mg GAE/g LW). These results indicate that solvent choice strongly influences the recovery of polyphenolic compounds from garlic, with EtOH:PBS extraction yielding markedly higher TPC than aqueous PBS.

Taken together, the higher activity observed in the DPPH and ABTS assays corresponds to the greater polyphenol content of the EtOH:PBS extract, whereas the PBS extract showed stronger ORAC activity despite containing very low levels of total phenolics, suggesting the presence of non-polyphenolic antioxidant compounds.

### 3.4. Quantification of the Sulfur-Containing Compound Alliin

**Figure 3:**
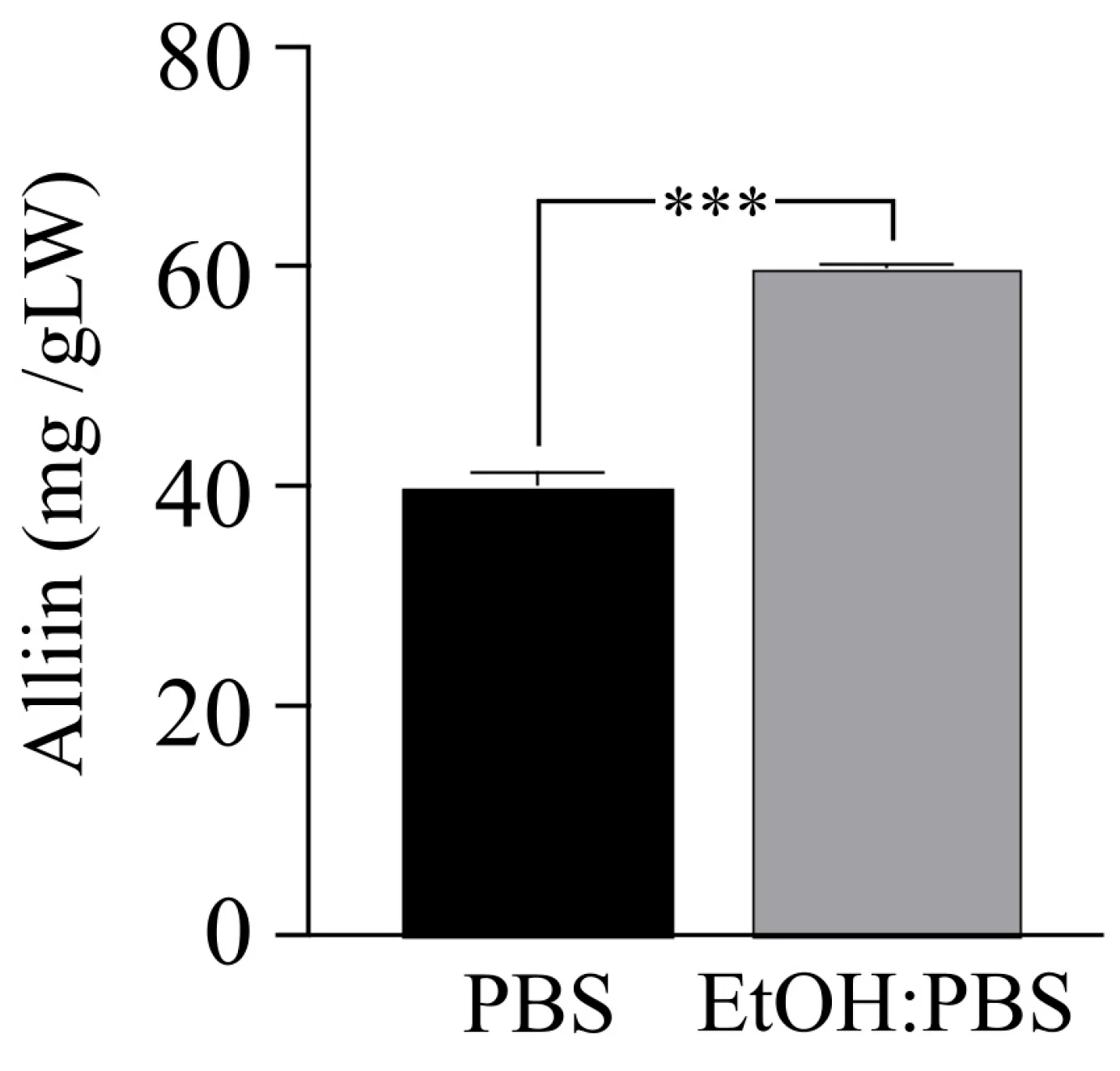
Quantification of alliin by HPLC in the PBS and EtOH:PBS extracts. Data are presented as mean ± SD from three analytical runs performed using the same extract preparation (*n* = 3 analytical runs). Each analytical run included technical triplicates. LW, lyophilized garlic weight. Statistical differences were assessed by Welch’s *t*-tests. Asterisks indicate statistically significant differences between extracts: *** *p* < 0.001.

The higher ORAC antioxidant capacity observed in the PBS extract is likely attributable to water-soluble sulfur compounds naturally abundant in garlic, such as alliin. To assess its contribution, we quantified alliin levels in both extracts using HPLC-UV. Alliin was consistently detected at a retention time of 5.56 min. Quantification revealed significant differences between the extracts; unexpectedly, the EtOH:PBS extract contained a higher concentration (60.05 ± 0.002 mg/g LW) than the PBS extract (41.12 ± 0.097 mg/g LW) (Figure 3). This finding suggests that the higher ORAC activity in the PBS extract may be driven by compounds other than alliin, highlighting the influence of solvent polarity on the recovery of sulfur-containing antioxidants. However, alliin content does not indicate the extent of allicin formation.

### 3.5. UPLC-ESI-TQ-MS/MS Analysis of the PBS and EtOH:PBS Extracts

To gain further insight into the chemical profiles of the extracts, we performed untargeted MS analysis of *m*/*z* signals to compare the PBS and EtOH:PBS extracts, followed by MS/MS analysis of selected relevant precursor ions.

**Figure 4:**
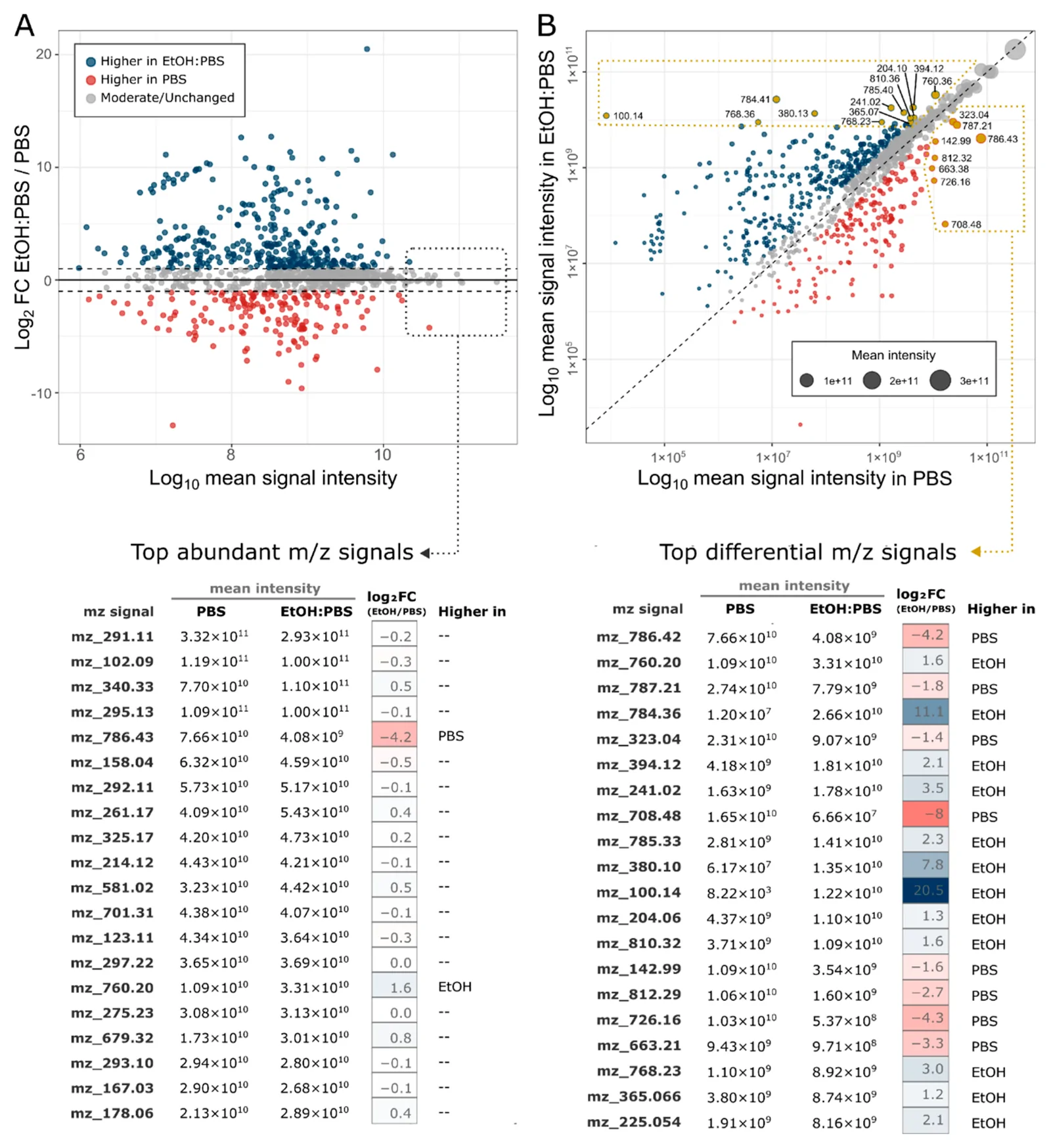
Comparative analysis of *m*/*z* signals detected in the PBS and EtOH:PBS extracts of *Allium sativum*. (**A**) MA plot showing the relationship between log_10_ mean signal intensity and log_2_ fold change, calculated as log_2_(EtOH:PBS/PBS). Positive Log_2_FC values indicate *m*/*z* signals with higher abundance in the EtOH:PBS extract, whereas negative values indicate signals with higher abundance in PBS extracts. Signals with |log_2_FC| > 1 were considered differentially abundant. (**B**) Scatter plot comparing log_10_ mean signal intensities between PBS and EtOH:PBS extracts. Points above the diagonal show signals more abundant in EtOH:PBS, while points below the diagonal show signals more abundant in the PBS extract. Bottom tables summarize the top abundant *m*/*z* signals and the top differential *m*/*z* signals ranked by mean intensity.

The distribution of log10-transformed mean intensities and log2 fold changes of the detected *m*/*z* signals showed substantial overlap between PBS and EtOH:PBS extracts, together with clear solvent-dependent differences in signal abundance (Figure 4). In total, 949 intensity-based *m*/*z* signals were detected, of which 455 (47.9%) showed relatively low variation between extraction conditions, defined as absolute log2 fold change values below 1. The remaining 494 signals (52.1%) displayed stronger differences between the extracts. Among these differentially abundant signals, 166 (17.5% of the total) were more abundant in the PBS extract, whereas 328 (34.6% of the total) were more abundant in the EtOH:PBS extract.

To evaluate the contribution of the most abundant signals, the top 20 *m*/*z* signals ranked by mean intensity were selected. Together, these signals accounted for 42.3% of the total cumulative intensity. Among them, 18 showed similar abundance between the PBS and the EtOH:PBS extracts (Figure 4A), with *m*/*z* 291.11 representing the most intense signal overall. Only two highly abundant signals exceeded the absolute log_2_ fold-change threshold (|log_2_FC| > 1): *m*/*z* 786.42, which was more abundant in the PBS extract, and *m*/*z* 760.20, which was more abundant in the EtOH:PBS extract. These results indicate that the most abundant signals were largely shared between extraction conditions, suggesting a common core of dominant *m*/*z* signals.

To further examine the most relevant signals differing between the extracts, we analyzed the top 20 intensity-based differential *m*/*z* signals, defined as those with absolute |log_2_FC| > 1. These signals accounted for 40.2% of the cumulative intensity of all differential signals and 7.4% of the total cumulative intensity. This subset showed a slightly uneven distribution between extraction conditions, with 12 *m*/*z* signals enriched in the EtOH:PBS extracts and 8 enriched in the PBS extracts (Figure 4B). Thus, among the most intense differential signals, both extraction conditions contributed to the observed differences in signal abundance between extracts.

Putative metabolite annotations were assigned to selected abundant and differential LC-MS features by integrating precursor *m*/*z* information, retention time (RT), theoretical molecular mass, daughter-ion fragmentation patterns, MS/MS spectral similarity searches against MoNA, MassBank, and GNPS, complementary information from FooDB, and evidence from published references. Based on this strategy, MS/MS analysis of selected abundant and differential features supported the putative annotation of several garlic-associated metabolites, including organosulfur compounds, amino acid-related metabolites, and phosphocholine-containing lipids (Table 3). Among the putatively annotated organosulfur compounds, the feature at m/z 178.06 was tentatively assigned to (+)-L-alliin, whereas the highly abundant feature at *m*/*z* 291.11 was tentatively annotated as γ-glutamyl-S-allylcysteine, which represented the most intense annotated feature and was detected at high abundance in both extraction conditions. Additional features were putatively assigned to sulfur-containing metabolites, including γ-glutamyl-S-methylcysteine, L-γ-glutamyl-S-allylthio-L-cysteine, and γ-L-glutamyl-S-(2-carboxy-1-propyl)cysteinylglycine. Some of these putatively annotated features showed clear solvent-dependent differences, with the feature tentatively assigned to L-γ-glutamyl-S-allylthio-L-cysteine enriched in PBS and the feature tentatively assigned to γ-L-glutamyl-S-(2-carboxy-1-propyl)cysteinylglycine enriched in the EtOH:PBS.

Two high-mass features, *m*/*z* 760.36 and *m*/*z* 786.43, showed fragmentation patterns consistent with phosphocholine-containing lipids, based on the diagnostic *m*/*z* 184.06 fragment. The former was more abundant in the EtOH:PBS extracts, whereas the latter was more abundant in the PBS extracts. Because the MS/MS information supported the lipid class but not the exact acyl-chain composition, these features were conservatively annotated as phosphatidylcholine-like or phosphocholine-containing lipids. Overall, the putatively annotated features suggest that both extracts share a core group of garlic-related organosulfur signals, while selected sulfur-containing and lipid-like features contribute to the solvent-dependent chemical differences observed between extracts.

### 3.6. Bacteriostatic Activity of the PBS and EtOH:PBS Extracts

**Figure 5:**
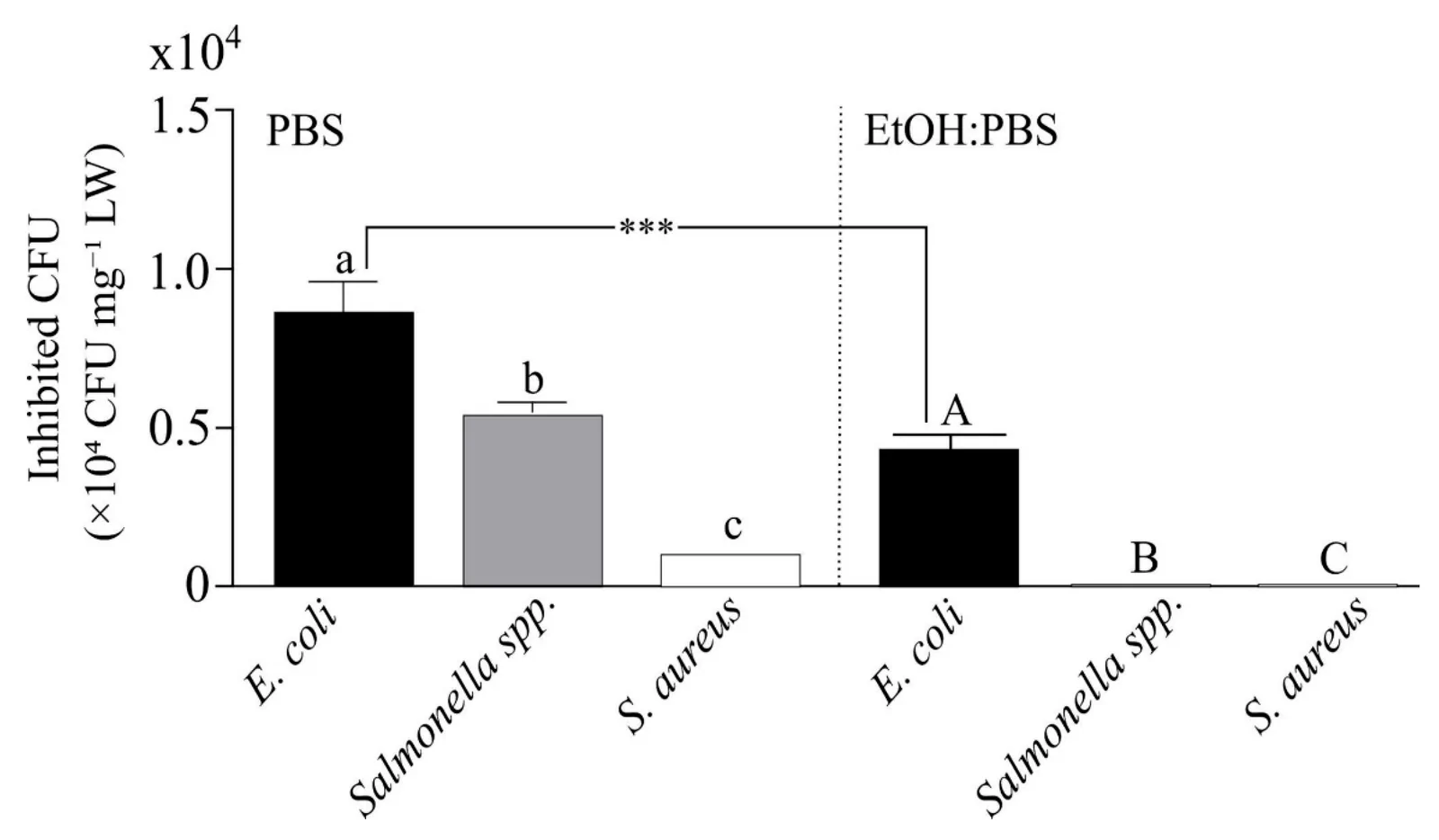
PBS and EtOH:PBS extracts differ in their antimicrobial activity across foodborne bacteria. Antimicrobial activity of PBS and EtOH:PBS extracts expressed as the number of colony-forming units (CFU) inhibited per mg of lyophilized extract (LW). Data are presented as mean ± SD from three separate microbiological assay runs performed using the same extract preparation for each solvent condition (*n* = 3). Technical triplicates were performed within each run and averaged before analysis. For each panel (PBS and EtOH:PBS), statistical differences among bacteria within the same extract were evaluated using a separate one-way ANOVA followed by Tukey’s HSD test. Compact letter display (CLD) indicates significant differences among bacteria within the same extract (*p* < 0.01). For the same bacterium, differences between extracts (PBS vs. EtOH:PBS) were assessed with pairwise *t*-tests and are indicated by asterisks (***, *p* < 0.001).

Garlic extracts are well known for their antimicrobial properties, which have been extensively studied using ethanolic and aqueous extracts, but not with PBS. To further characterize this activity, we evaluated the antimicrobial effects of freeze-dried PBS and EtOH:PBS extracts against *E. coli*, *Salmonella* spp., and *S. aureus*, expressed as colony-forming units (CFU) inhibited per mg of extract (Figure 5). Log-reduction (LR) analysis confirmed a bacteriostatic, non-bactericidal, effect for PBS: LR = 2.00 for *E. coli*, 1.95 for *Salmonella* spp., and 1.62 for *S. aureus*. The EtOH:PBS extract produced a limited effect (maximum LR = 1.46 against *E. coli*) and showed no inhibition of *Salmonella* spp. or *S. aureus*. Consistent with these trends, across the tested bacterial species, the PBS extract exhibited lower minimum inhibitory concentration (MIC) 15 mg/mL for *E. coli*, 30 mg/mL for *Salmonella* spp., and 60 mg/mL for *S. aureus*, whereas the EtOH:PBS extract required higher concentrations to approach comparable inhibition.

### 3.7. Effects of the PBS Extract and Alliin on Antioxidant Gene Expression in LPS-Stimulated 3T3-L1 Adipocytes

**Figure 6:**
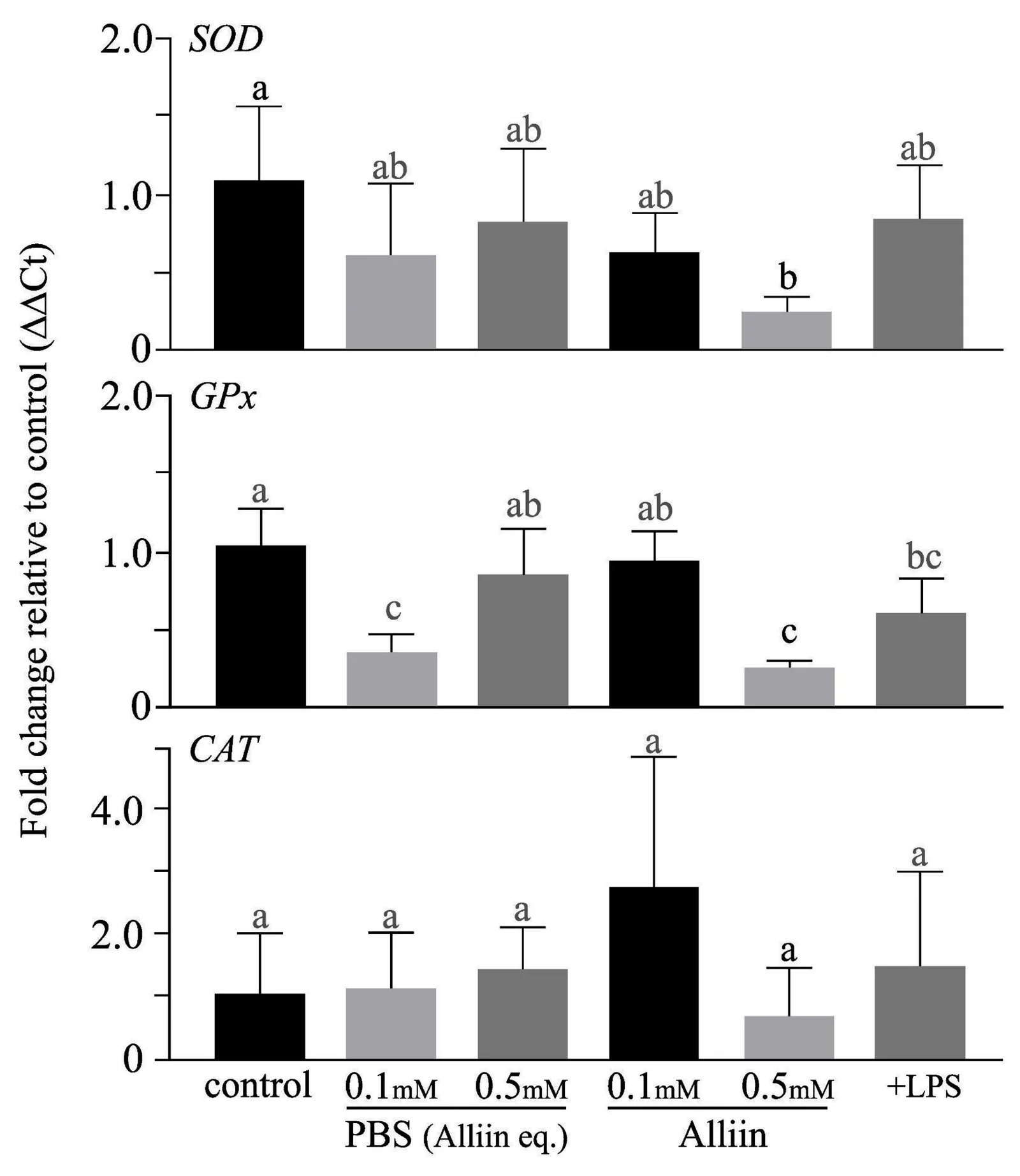
Effect of PBS extract and alliin on antioxidant gene expression in LPS-stimulated 3T3-L1 adipocytes. Experimental groups included untreated control cells, LPS-treated cells (+LPS), and LPS-treated cells exposed to either the PBS extract at alliin-equivalent concentrations of 0.1 or 0.5 mM or pure alliin at 0.1 or 0.5 mM. Relative expression of SOD, GPx, and CAT was quantified by RT-qPCR, normalized to *Rps18s*, and calculated using the 2^−ΔΔCt^ method. Data are presented as mean ± SD from three pooled samples per condition generated within a single cell-culture experiment (*n* = 3); each pooled sample comprised three separately treated wells. RT-qPCR was performed in technical duplicate and averaged before analysis. Statistical differences were determined by one-way ANOVA followed by Tukey’s HSD test (*p* < 0.05). Different letters in the compact letter display indicate statistically significant differences among groups.

To build on the antioxidant profile observed for the PBS extract, we investigated its modulation of oxidative gene expression and LPS-induced cytokine signaling in differentiated 3T3-L1 adipocytes. Differentiated 3T3-L1 adipocytes possess an active oxidative metabolism, making them suitable for analyzing cellular responses to oxidative stress. To assess the potential antioxidant effects of the PBS extract at the cellular level, we induced oxidative and inflammatory conditions by stimulating the cells with LPS, which is known to increase ROS production and impair antioxidant defenses (Figure 6). Treatment with LPS alone did not significantly alter *SOD* expression but caused a marked reduction in *GPx.* For *SOD*, a significant reduction was observed only with 0.5 mM alliin treatment, whereas the PBS extract did not produce this effect, even at an equivalent alliin concentration. In contrast, *GPx* expression decreased significantly with the PBS extract at an alliin-equivalent concentration of 0.1 mM and with 0.5 mM alliin. *CAT* expression was also evaluated; however, no significant differences were observed among the treatments. Overall, these results indicate a selective modulation of antioxidant genes by the PBS extract and alliin, with *GPx* being more sensitive than *SOD* under these conditions.

### 3.8. Exploratory Cytokine Profile Following Treatment with the PBS Extract or Alliin in LPS-Stimulated 3T3-L1 Adipocytes

**Figure 7:**
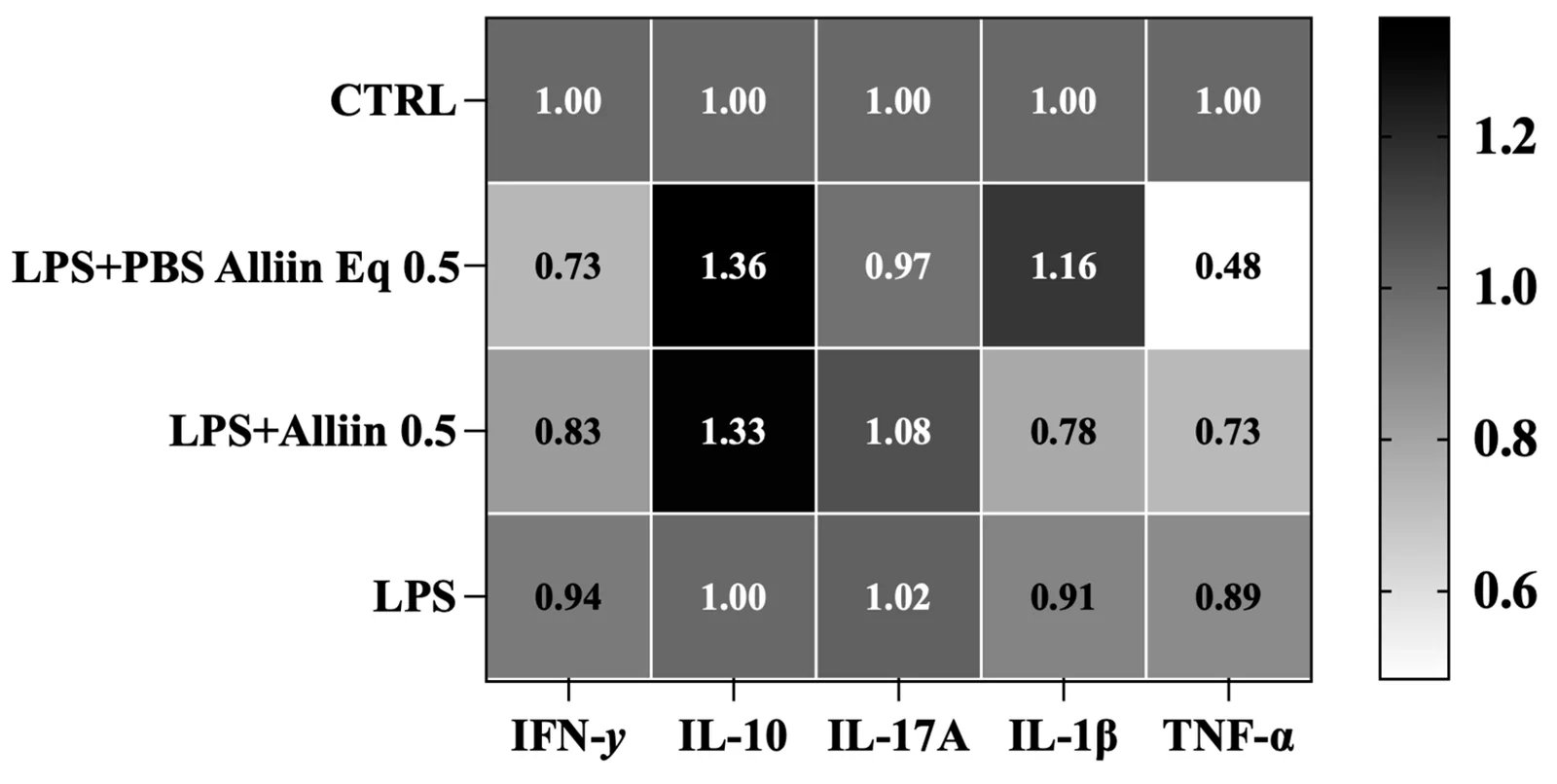
Exploratory cytokine profiles following treatment with the PBS extract or alliin in LPS-stimulated 3T3-L1 adipocytes. Differentiated 3T3-L1 adipocytes treated with the PBS extract at an alliin-equivalent concentration of 0.5 mM (PBS Alliin Eq 0.5 + LPS) or pure alliin at 0.5 mM (Alliin 0.5 + LPS), compared with untreated cells (CTRL) and the LPS control group (LPS). Heatmap showing relative fluorescence values for each cytokine (columns) across treatments (rows), normalized to the untreated control (CTRL), set to 1.00. Numbers within tiles represent fold-change relative to CTRL. Darker shades indicate higher relative fluorescence, while lighter shades indicate lower relative fluorescence. The analysis was exploratory and semi-quantitative, and no correction for multiple comparisons was applied. Cytokine measurements were obtained from three pooled cell-culture supernatants per condition within a single cell culture experiment. Each pooled sample was generated by combining the supernatants from three separately treated wells. Each pooled sample was analyzed once with no technical replicates, and the fluorescence values obtained were averaged.

Following the antioxidant gene expression analysis, we explored the cytokine profiles of differentiated 3T3-L1 adipocytes stimulated with LPS and treated with either the PBS extract at an alliin-equivalent concentration of 0.5 mM or pure alliin at 0.5 mM. Relative fluorescence signals for IL-1β, IFN-γ, IL-10, IL-17A, and TNF-α were normalized to the untreated control group (CTRL, set at 1.00) and expressed as fold change (Figure 7). Descriptive differences in cytokine profiles were observed among the experimental groups. IFN-γ signals were lower following treatment with either the PBS extract or pure alliin, whereas pure alliin produced a moderate increase in IL-10. TNF-α showed contrasting patterns between treatments, with a lower signal following alliin treatment and a higher signal following treatment with the PBS extract. IL-17A remained comparatively stable across conditions, while the PBS extract and pure alliin showed different descriptive patterns for IL-1β. Overall, these observations suggest that the PBS extract and pure alliin may differentially influence cytokine profiles under the experimental conditions evaluated. However, because the analysis was exploratory and semi-quantitative and no correction for multiple comparisons was applied across cytokines or treatment groups, the observed patterns should be considered descriptive and hypothesis-generating rather than confirmatory evidence of treatment effects.

## 4. Discussion

PBS extraction provides a biologically compatible route to garlic-derived preparations that can be directly applied in downstream assays without additional solvent-removal or preprocessing steps [17,18]. However, this practical advantage may come with an important chemical trade-off, as the spectrum of compounds recovered in PBS can differ markedly from that obtained with hydroalcoholic solvents [16,24]. In this study, comparison of PBS and EtOH:PBS extracts showed that extraction solvent not only influenced the chemical profile of the preparations but also shaped their bioactive properties, particularly peroxyl radical-scavenging capacity and antimicrobial activity.

Both extraction systems recovered comparable amounts of lyophilized material and shared a dominant core of abundant *m*/*z* signals, indicating that the PBS and EtOH:PBS extraction systems captured overlapping components of the detected garlic chemical profile. This shared chemical background, however, was accompanied by solvent-dependent shifts in specific features, including signals putatively annotated as organosulfur compounds, phosphocholine-containing features, as well as differences total polyphenol content and HPLC-quantified alliin levels. Thus, solvent choice did not generate two entirely separate chemical profiles, but rather reshaped the relative abundance of detected signals within a shared garlic-derived chemical profile (Figure 4 and Table 3).

Importantly, the biological behavior of the extracts was not simply predicted by either total polyphenol or alliin abundance (Figure 2 and Figure 3). The EtOH:PBS extract, which contained markedly higher levels of total polyphenols, showed stronger radical-scavenging activity in the DPPH and ABTS assays (Figure 1). In contrast, the PBS extract, despite its lower polyphenol and alliin content, showed greater peroxyl radical-scavenging capacity in the ORAC assay and stronger bacteriostatic activity across the tested bacterial strains (Figure 5). These results suggest that the bioactivity of the PBS extract may arise from the combined contribution of multiple water-compatible compounds rather than from a single dominant marker compound.

Garlic extracts are consistently reported to display antioxidant activity, a property that contributes to several downstream biological effects, including cytoprotective and anti-inflammatory responses [17]. In our study, the contrasting results among antioxidant assays are consistent with their different chemical principles. DPPH and ABTS assays largely capture general radical-scavenging and electron-transfer capacity, which may explain the higher activity of the EtOH:PBS extract in association with its higher polyphenol content [16,17,34]. ORAC, by contrast, evaluates the time-dependent inhibition of peroxyl-radical-induced oxidation, a process that is particularly relevant to biological oxidative damage [13,15]. The stronger ORAC response of the PBS extract therefore points to antioxidant properties that may be more closely related to physiological redox conditions.

Previous studies have shown that aqueous garlic extracts often display lower antioxidant activity than ethanolic or hydroethanolic extracts in DPPH and ABTS assays, partly due to their lower phenolic content [17]. Our results agree with this trend for DPPH and ABTS, but not for ORAC. This divergence indicates that the antioxidant potential of the PBS extract cannot be explained by total polyphenols alone. Likewise, alliin content does not fully account for the observed ORAC activity, since alliin was lower in the PBS extract than in the EtOH:PBS extract. The compounds responsible for this response remain to be resolved, but selected features putatively annotated as γ-glutamyl peptides, and other organosulfur derivatives, together with water-compatible compounds may contribute to the antioxidant profile [20,33]. This interpretation is consistent with previous reports linking the antioxidant activity of garlic preparations to both phenolic and sulfur-containing compounds, as well as with observations associating ORAC activity with organosulfur compound composition [21,27,35,36]. Because all UPLC-MS annotations were putative and only selected precursor ions were evaluated by MS/MS, specific relationships between individual putatively annotated compounds and ORAC activity cannot yet be established.

To further examine the biological relevance of this antioxidant behavior, we focused the cellular experiments on the PBS extract, because it showed the highest ORAC activity and was directly compatible with cell-culture conditions. In LPS-stimulated 3T3-L1 adipocytes, the PBS extract selectively modulated oxidative stress–related genes, with GPx showing greater sensitivity than SOD or CAT. The reduction in GPx expression at the lower alliin-equivalent concentration may reflect a lower intracellular peroxide burden, potentially associated with the radical-scavenging capacity of compounds present in the extract. The comparatively mild SOD response is consistent with previous evidence that antioxidant enzymes do not respond uniformly in differentiated 3T3-L1 cells, where SOD may show limited inducibility under inflammatory stress [37,38]. Pure alliin produced a related pattern only at the higher concentration, suggesting that additional compounds present in the PBS extract may contribute to the cellular response.

This interpretation is consistent with the idea that garlic-derived γ-glutamyl peptides and related sulfur compounds may influence intracellular thiol metabolism and redox-sensitive pathways, including the Nrf2–GPx axis [39,40]. However, this mechanism remains speculative and requires direct experimental validation. The absence of a clear CAT response may reflect the more compartmentalized regulation of catalase, which primarily detoxifies high H_2_O_2_ loads in peroxisomes and may be less transcriptionally responsive under the conditions tested [41]. Together, these results support a selective, rather than global, modulation of antioxidant defenses by the PBS extract.

Differentiated 3T3-L1 adipocytes are relevant for studying low-grade inflammation because mature adipocytes contribute to the inflammatory tone of adipose tissue [42]. However, they often respond less strongly to acute LPS stimulation than preadipocytes, and several cytokines, including TNF-α and IL-1β, may peak during the first hours of stimulation and decline by 24 h [43,44,45,46]. Under this framework, the modest cytokine changes observed here are consistent with the expected inflammatory kinetics of mature adipocytes. The PBS extract and pure alliin showed partially overlapping effects for IFN-γ, IL-10, IL-17A, and TNF-α, but differed in their effects on IL-1β, suggesting that the extract may exert a more complex immunomodulatory influence than alliin alone. Previous work showing that alliin attenuates LPS-induced inflammation in 3T3-L1 adipocytes through modulation of ERK1/2, IL-6, and CCL2 further supports the relevance of garlic sulfur compounds in adipocyte inflammatory signaling [3,39,47]. Nevertheless, the cytokine analysis has important limitations. Because the fluorescence measurements were semi-quantitative and multiple cytokines and treatment conditions were examined without correction for multiple comparisons, the probability of type I error may be increased. Accordingly, the observed cytokine patterns should be interpreted as exploratory and hypothesis-generating rather than as confirmatory evidence of immunomodulatory effects or a defined mechanism. Future studies should include independent biological replication, predefined cytokine endpoints, adequate statistical power, and an appropriate multiple-comparison correction, such as control of the false discovery rate or family-wise error rate.

In addition to its antioxidant profile, the PBS extract showed stronger bacteriostatic activity than the EtOH:PBS extract across the tested bacterial strains. Garlic antimicrobial activity is commonly attributed to organosulfur compounds such as allicin, diallyl disulfide, and diallyl trisulfide, which can disrupt bacterial membranes, modify protein thiols, inhibit essential enzymes, and alter oxidative balance [8,48]. However, allicin, diallyl disulfide, and diallyl trisulfide were not directly quantified in the present extracts. Therefore, their specific contribution to the observed bacteriostatic activity cannot be established and remains a literature-supported hypothesis. Although the log-reduction values observed here did not reach the threshold for bactericidal activity, they support a clear bacteriostatic effect. The greater activity of the PBS extract suggests that aqueous, buffered conditions may better preserve or recover antimicrobial sulfur-containing compounds, whereas hydroalcoholic extraction systems, despite increasing phenolic recovery, may not necessarily enhance antibacterial activity [17,35].

This distinction is relevant because the tested bacteria, including *E. coli*, *Salmonella* spp., and *S. aureus*, are associated with intestinal imbalance, inflammation, and foodborne infection. In this context, the bacteriostatic activity of the PBS extract may be biologically meaningful, particularly when considered together with its antioxidant and exploratory immunomodulatory effects [49,50,51,52]. Moreover, previous reports of synergy between allicin and conventional antibiotics suggest that garlic-derived sulfur compounds may have value as complementary antimicrobial agents [10].

Overall, our findings show that the PBS extraction system does not simply represent a weaker aqueous alternative to hydroalcoholic or ethanolic extraction systems. Rather, the PBS extract exhibited a distinct functional profile characterized by lower polyphenol recovery, lower alliin content, stronger ORAC activity, greater bacteriostatic effects, and selective modulation of cellular redox and inflammatory markers. Because the PBS extraction system is biocompatible and avoids solvent-removal steps that may compromise labile or volatile compounds, it offers a practical extraction system for direct biological evaluation [17,24,53]. Further studies are needed to confirm the identities of the putatively annotated features, determine which compounds are responsible for these activities, assess their stability, and define the experimental or biomedical contexts in which PBS extraction provides an advantage over conventional solvent systems.

## 5. Conclusions

Our findings show that PBS extraction does not merely provide a biocompatible alternative to EtOH:PBS extraction but produces an extract with a distinct functional profile. Although both extraction systems showed substantial overlap in their dominant *m*/*z* signals, solvent-dependent differences in signal abundance and selected putatively annotated features were observed alongside distinct biological outcomes. The PBS extract, despite containing lower levels of total polyphenols and alliin, showed stronger ORAC activity and greater bacteriostatic effects than the EtOH:PBS extract, indicating that its bioactivity is not explained by conventional marker compounds alone.

This functional profile was further reflected in LPS-stimulated 3T3-L1 adipocytes, where the PBS extract selectively modulated oxidative stress–related gene expression, particularly GPx, and produced exploratory changes in cytokine patterns. Together, these responses suggest that the PBS extract acts at the intersection of redox regulation, microbial growth control, and inflammatory signaling, likely through the combined contribution of multiple water-compatible compounds.

Overall, PBS extraction should not be viewed as a chemically weaker aqueous substitute for hydroalcoholic or ethanolic extraction, but as a low-processing and biologically compatible extraction system that reveals a distinct dimension of garlic bioactivity. Future work should move from putative annotation and association toward structural confirmation and mechanism by identifying the metabolites or metabolite combinations responsible for these effects, determining their stability, and testing how they interact with cellular redox and inflammatory pathways over time.

## Figures and Tables

**Table 1:** Primers used for qRT-PCR.

Gene	Forward	Reverse	Product (bp)	Temp. (°C)
*Rps18s*	AGAAACGGCTACCACATCCA	CCCTCCAATGGATCCTTCGTT	158	56
*CAT*	GCAGATACCTGTGAACTGTC	GTAGAATGTCCGCACCTGAG	229	63
*SOD*	TGGTGGTCCATGAGAAACAA	GTTTACTGCGCAATCCCAAT	115	61
*GPx*	GTCCACCGTCTTATGCCTTCT	TCTGCAGATCGTTCATCTCG	152	60

*Rps18s*: ribosomal protein S18; *CAT*: catalase; *SOD:* superoxide dismutase; *GPx*: glutathione peroxidase; bp: Product size in base pairs; Temp.: annealing temperature (°C).

**Table 2:** Extraction yield of the PBS and EtOH:PBS extracts.

Solvent	PBS	EtOH:PBS
Initial weight (g)	51.5 ± 0.7	52.7 ± 0.2
Initial volume (mL)	500	500
Extract LW (g)	12.7 ± 0.6	9.4 ± 1.3
Extraction yield (%)	24.7 ± 4.1	17.9 ± 2.6

PBS: phosphate-buffered saline; EtOH:PBS: 50% EtOH in PBS *v*/*v*; Extract LW: lyophilized extract weight; Performance: extraction yield expressed as percentage (mean ± SD).

**Table 3:** Putative annotation, fragmentation patterns, and relative abundance of selected features detected by UPLC-ESI-TQ-MS/MS in the PBS and EtOH:PBS extracts.

Putative Annotation	Formula (Mass Da)	RT (min)	Precursor *m*/*z* ([M + H]+)	Fragments	PubChem CID	Platform/ Reference	PBS AUC	EtOH:PBS AUC
(+)-L-Alliin	C6H11NO3S (177.22)	0.51	178.0605	88.02, 74.00, 119.00	9576089	MassBank Molina-Calle et al., 2017 [20]	1.09 × 10^10^	1.78 × 10^10^
γ-glutamyl-S-methylcysteine	C9H16N2O5S (264.296)	0.63	265.09	88.02, 119.00, 74.00, 84.00,	11043643	FooDB	1.02 × 10^10^	9.09 × 10^9^
γ-Glutamyl-S-allylcysteine	C11H18N2O5S (290.339)	1.55–1.82	291.11	73.00, 130.00	11346811	MassBank; Molina-Calle et al., 2017 [20]	3.32 × 10^11^	2.93 × 10^11^
γ-Glutamylphenylalanine	C14H18N2O5 (294.30)	2.44	293.1035	38.16, 54.82, 55.03, 55.24, 58.12, 59.32, 67.12, 73.09, 74.99, 83.98, 84.19, 85.03, 100.07, 101.05, 117.92, 118.27, 130.21, 131.26, 131.97, 146.16, 147.07, 153.18, 164.07, 170.11, 184.37, 194.41, 229.18, 230.23	111299	MoNA; Molino et al., 2021 [33]	2.05 × 10^11^	1.40 × 10^11^
γ-Glutamyl-S-allylthio-L-cysteine	C11H18N2O5S2 (322.065)	3.25	323.036	41.09, 41.29, 42.72, 55.97, 56.18, 73.03, 84.11, 88.12, 105.93, 107.01, 120.06, 120.2, 130.05, 153.22, 185.97, 194.12	131752387	MassBank MoNA Molina-Calle et al., 2017 [20]	2.13 × 10^10^	2.47 × 10^8^
γ-Glutamyl-S- (2-carboxy-1-propyl) cysteinylglycine	C14H23N3O8S (393.1205)	1.09	394.122	41.02, 55.02, 68.96, 84.18, 91.18, 99.06, 111.7, 115.98, 119.11, 129.23, 130.12, 143.91, 144.18, 145.00, 153.36, 171.63, 187.87, 201.8, 230.06,	20140672	FooDB	3.89 × 10^9^	8.04 × 10^9^
Phosphatidylcholine like lipid	C43H64NO10P (785.942)	17.07	786.4265	184.06, 229.31, 417.5, 548.33	NA	Lipidmaps; MoNA Molino et al., 2021 [33] (based on the diagnostic *m*/*z* 184 fragment)	7.66 × 10^10^	4.08 × 10^9^
Phosphocholine-containing lipid—PC(16:0/18:1)-like	C42H82NO8P (759.577)	16.1	760.3575	86.15, 183.73, 184.00, 184.27, 759.67	5313964	Lipidmaps; Molino et al., 2021 [33] (based on the diagnostic *m*/*z* 184 fragment)	1.59 × 10^8^	7.05 × 10^9^

RT: retention time; *m*/*z*: mass-to-charge ratio; CID: PubChem compound identifier; PBS: phosphate-buffered saline; EtOH:PBS: 50% ethanol in PBS (*v*/*v*); AUC: area under the curve; NA: not applicable.

## Data Availability

The original contributions presented in this study are included in the article/Supplementary Materials. Further inquiries can be directed to the corresponding author(s).

## Supplementary Materials

The following supporting information can be downloaded at: https://www.mdpi.com/article/10.3390/metabo16080572/s1, Supplementary S1: Standard Curves for Antioxidant and Polyphenol Assays; Figure S1: Calibration curves for antioxidant and polyphenol assays; Supplementary S2: Calculation of alliin-equivalent concentrations for cell culture treatments.

## Abbreviations

The following abbreviations are used in this manuscript:

PBS	Phosphate-Buffered Saline
EtOH	Ethanol
DPPH	2,2-Diphenyl-1-picrylhydrazyl
ABTS	2,2′-Azino-bis(3-ethylbenzothiazoline-6-sulfonic acid)
ORAC	Oxygen Radical Absorbance Capacity
HPLC	High-Performance Liquid Chromatography
UPLC-ESI-TQ-MS/MS	Ultra-Performance Liquid Chromatography Coupled to Electrospray Ionization Tandem Triple Quadrupole Mass Spectrometry
CFU	Colony-Forming Units
MIC	Minimum Inhibitory Concentration
GPx	Glutathione Peroxidase
SOD	Superoxide Dismutase
IL-10	Interleukin-10
TNF-α	Tumor Necrosis Factor Alpha
IFN-γ	Interferon Gamma
LPS	Lipopolysaccharide
DMEM	Dulbecco’s Modified Eagle Medium
FBS	Fetal Bovine Serum
IBMX	3-Isobutyl-1-methylxanthine
ROS	Reactive Oxygen Species
