# Supplementary material for: Bioactive Potential of “Jaspeado Garlic” (Allium sativum) from Northwestern Mexico: In Vitro Antioxidant and Bacteriostatic Effects
Source: Metabolites. 2026 Aug 12;16(8):572. doi: 10.3390/metabo16080572 (PMC13515696; doi:10.3390/metabo16080572)
Supplement: Supplementary file 1 [file metabolites-16-00572-s001.zip › metabolites-4011512-supplementary.pdf]

metabolites

MDPI

Supplementary Material

Supplementary S1

S.1 Standard Curves for Antioxidant and Polyphenol Assays

Calibration curves used for the quantification of antioxidant activity by DPPH, ABTS, and ORAC assays, and for the determination of total polyphenol content. Linear regression models are shown for DPPH radical inhibition (% vs. µM Trolox), ABTS•⁺ radical inhibition (% vs. µM Trolox), ORAC (net AUC vs. µM Trolox), and total polyphenols measured by the Folin–Ciocalteu method (absorbance vs. µg gallic acid equivalents per mL, GAE/mL). Each panel displays the regression equation and the coefficient of determination (R^2).

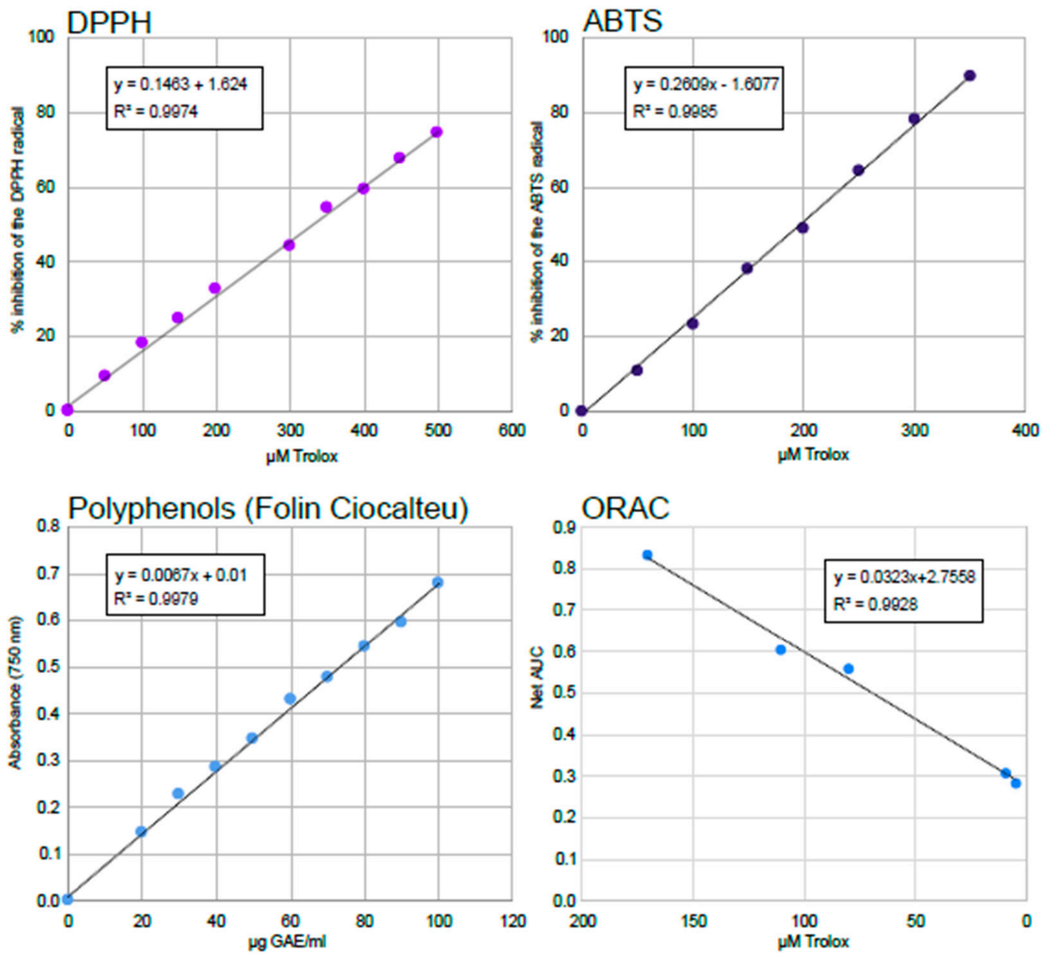

***Figure S1**. Calibration curves for antioxidant and polyphenol assays.*

Supplementary S2

S.2 Calculation of alliin-equivalent concentrations for cell culture treatments

The doses applied were selected based on prior work in 3T3-L1 adipocytes showing that alliin is active and non-toxic within the 0.1 to 1.0 mM range (Quintero-Fabián et al., 2013; Quintero, 2013 thesis). The 0.1 mM dose represents the minimal effective concentration, while 0.5 mM corresponds to a higher, yet previously documented, non-cytotoxic concentration.

For the cell treatment groups, only the PBS extract was used due to its intrinsic biocompatibility and suitability for direct application in cell culture. This extract also showed the highest antioxidant performance in the ORAC assay, which monitors the neutralization of physiologically relevant peroxyl radicals. Extract concentrations were standardized using alliin-equivalent dosing. The alliin content of the PBS extract was quantified by HPLC (41.12 ± 0.097 mg/g LW). Using this value, the target concentrations of 0.1 mM and 0.5 mM alliin were converted to their equivalent extract masses based on the molecular weight of alliin (177.2 g/mol), resulting in final working concentrations of 0.43 mg/mL and 2.15 mg/mL of extract.
